# Supplementary material for: CYR61 delivery promotes angiogenesis during bone fracture repair
Source: NPJ Regen Med. 2025 Apr 22;10:20. doi: 10.1038/s41536-025-00398-y (PMC12015299; doi:10.1038/s41536-025-00398-y)
Supplement: Supplementary file 1 — Supplementary Information [file 41536_2025_398_MOESM1_ESM.pdf]

Supplementary Information

CYR61 delivery promotes angiogenesis during bone fracture repair

Annemarie Lang, Emily A. Eastburn, Mousa Younesi, Madhura Nijsure, Carly Siciliano, Annapurna Pranatharthi Haran, Christopher J. Panebianco, Elizabeth Seidl, Rui Tang, Eben Alsberg, Nick J. Willett, Riccardo Gottardi, Dongeun Huh, Joel D. Boerckel

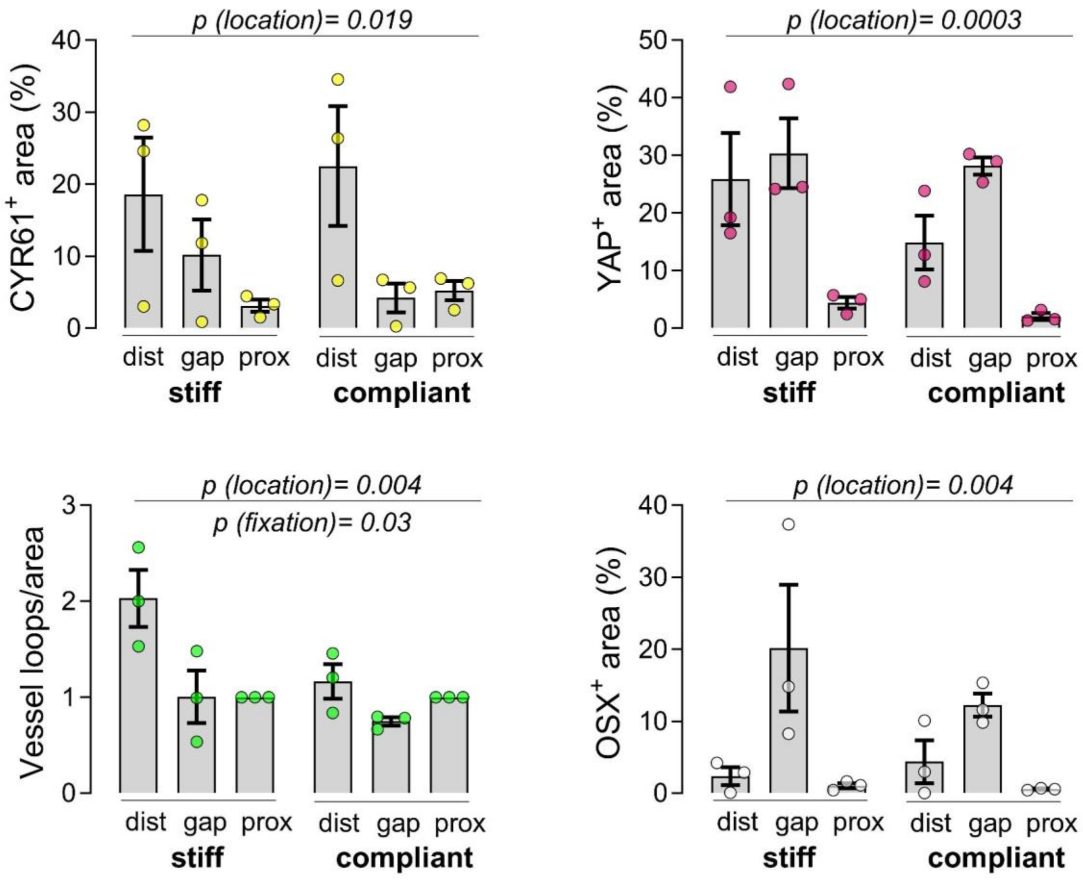

Supplementary Figure 1

Spatial expression of YAP, CCN1/CYR61, EMCN and OSX stiff vs. compliant fixation. Separated quantifications. Ordinary two-way ANOVA was performed to determine main effects of location (distal, gap, proximal) and fixation (stiff vs. compliant). Statistical significances are provided in graphs.

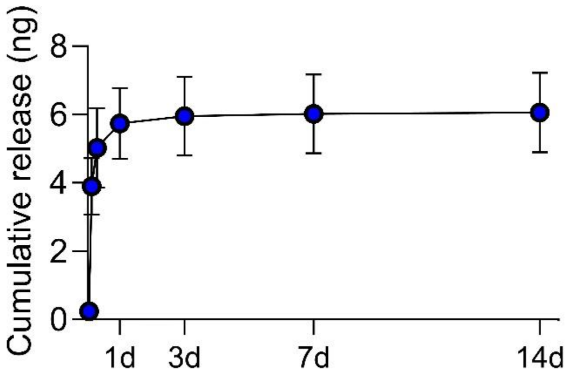

Supplementary Figure 2

CYR61 release kinetics from GelMA/fibrin scaffolds determined over 14d. 1 µg rhCYR61 was loaded into GelMA-fibrin scaffolds and release into 1 mL PBS was measured by ELISA over 14 days. Cumulative measured release averaged 6 ng (0.6% of total loaded protein).

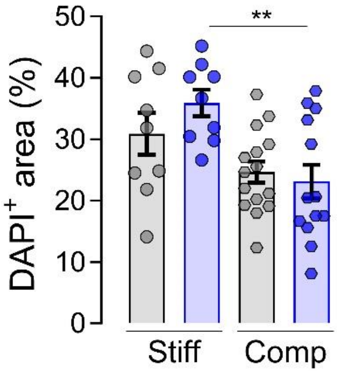

Supplementary Figure 3

Supporting data at 14 dpf. Quantification of DAPI in all samples undergoing EMCN/OSX and F4/80/ SOX9 staining (n= 8-14; duplicate per sample). Control = grey; CYR61 = blue
